# Quality of life impacts associated with comorbid insomnia and depression in adult population

**DOI:** 10.1007/s11136-024-03793-y

**Published:** 2024-09-26

**Authors:** Phuong Hong Le, Long Khanh-Dao Le, Shantha M.W. Rajaratnam, Cathrine Mihalopoulos

**Affiliations:** 1 Monash University Health Economics Group (MUHEG), School of Public Health and Preventative Medicine, Monash University, Melbourne, Australia; 2 School of Psychological Sciences and Turner Institute for Brain and Mental Health, Monash University, Clayton, Australia

**Keywords:** Comorbidity, Depression, Health-related quality of life, Insomnia, Utility score

## Abstract

**Purpose:**

Health-related quality of life (HRQoL) impacts of insomnia and depression (as separated entities) have been well investigated in previous studies. However, little is known about the effect of comorbid insomnia and depression on HRQoL. This study aimed to assess the impacts of insomnia and depression, in combination or alone, on HRQoL in Australian adults.

**Methods:**

Data used in this study were obtained from the large-scale longitudinal Household, Income and Labour Dynamics in Australia (HILDA) survey. Insomnia was defined using key insomnia criteria of DSM-V. Depression was based on validated cut-off points of the Mental Health Inventory-5 (MHI-5) (scores ≤ 62) in the base case analysis. HRQoL expressed as utility scores (ranging from 0 to 1) were measured using the Short-Form 6-Dimension (SF-6D) converted from the SF-36 and valued using an Australian scoring algorithm. Multi-level modelling was applied to assess the effect of insomnia and/or depression on utility scores.

**Results:**

The study analysed 30,972 observations from 10,324 individuals (age [mean ± SD]: 45.7 ± 16.5, female: 54.6%). The proportion of individuals with insomnia only, depression only, and comorbid insomnia and depression was 11.3%, 11.6%, and 8.2%, respectively. The interaction effect suggested the combined impact of insomnia and depression on health-related quality of life beyond the sum of their individual effects. Marginal mean difference in utility scores for insomnia only, depression only, and the comorbidity relative to no insomnia or depression was -0.058 (SE: 0.003, Cohen’s d: 0.420, small effect), -0.210 (SE: 0.003, Cohen’s d: 1.530, large effect), and -0.291 (SE: 0.004, Cohen’s d: 2.120, large effect), respectively.

**Conclusion:**

Comorbid depression and insomnia appear to have very large quality-of-life impacts. Furthermore, this is the first study that has estimated the magnitude of the impact of comorbid insomnia and depression on utility scores which can be utilised in future clinical or economic studies.

**Supplementary Information:**

The online version contains supplementary material available at 10.1007/s11136-024-03793-y.

## Abstract (plain language summary)

Insomnia and depression often occur together and have an evidence-based bidirectional relationship. The impairment of health-related quality of life (HRQoL) associated with insomnia or depression, as an individual effect, has been previously examined by several studies. However, the reduction in HRQoL associated with comorbid insomnia and depression has been understudied. With the use of representative longitudinal data containing a large sample size of 10,324 Australian adults, we found that insomnia and depression were associated with statistically significant reductions in health-related quality of life, whether occurring individually or concurrently. The effect of comorbid insomnia and depression on quality of life was significantly larger than the summative effect of insomnia and depression. This study provides new insights into the quality-of-life burden of insomnia and/or depression and emphasises the importance of addressing insomnia in adults with depression.

**Supplementary Information:**

The online version contains supplementary material available at 10.1007/s11136-024-03793-y.

## Introduction

Depression is one of the 25 leading causes contributing to the global health-related burden with a dramatic upward trend of prevalence and incidence rates in the last ten years [1]. Insomnia and depression often occur concurrently [2], with 41% of those with depression experiencing insomnia [3]. Moreover, depression and insomnia have a bidirectional relationship that has been investigated by several studies, especially in adult populations [4–6]. Insomnia has been found to be a risk factor that impairs depression treatments and predicts subsequent depression [4, 7–11]. Additionally, increasing evidence has shown that insomnia treatment may alleviate depressive symptoms in individuals with comorbid insomnia and depression [12–14]. Conversely, depression is also considered a risk factor for future insomnia [15].

Health-related quality of life (HRQoL) is a multidimensional concept that is widely used to capture the health status of individuals. The two main dimensions of HRQoL are usually physical and mental health [16]. Measuring HRQoL can provide new insights into the burden of preventable diseases, injuries, and disabilities [16]. HRQoL can be measured by different questionnaires such as the World Health Organization Quality-of-Life (WHO-Qol) [17] or the Short Form (SF)-36 [18]. HRQoL is usually expressed as utility scores commonly derived from HRQoL questionnaires that have preference-based scoring algorithms (commonly referred to as multi-attribute utility instruments - MAUIs). The scoring algorithms convert scores on the questionnaires to a scale of 0 to 1. A score of 1 denotes perfect health as measured by the questionnaire and 0 denotes death [19]. There can be health states measured on the questionnaires that have values that are less than 0 – these health states have been judged to be worse than death [20]. Commonly used MAUIs include the EQ-5D [21], the SF-6D – derived from the SF-12 or SF-36 [22], Health Utilities Index (HUI) [23], and the suite of Assessment of Quality of Life (AQoL) [24] questionnaires. Utility scores are also used to determine quality-adjusted life years (QALYs), a generic health outcome measure recommended in several guidelines for economic evaluations [25–27].

Impairment of HRQoL associated with insomnia or depression has been examined by previous studies. The mean utility scores for those with major depressive disorder (MDD) have been found to be significantly lower than the scores for those without mental disorders/symptoms (0.65 ± 0.28 versus 0.88 ± 0.17, effect size: -1.32, p-value < 0.001) (assessed with AQoL-4D) [28]. Individuals with chronic insomnia have been similarly found to have significantly lower utility scores compared to those without insomnia (0.63 versus 0.72 in the US, p-value < 0.001, 0.57 versus 0.67 in France, p-value < 0.001, and 0.67 versus 0.77 in Japan, p-values < 0.001) (all measured by SF-6D) [29]. However, these scores are not directly compared given that utilities derived from different MAUIs will result in different scores [30]. Furthermore, little is known about the interaction effect of both insomnia and depression on utility scores. This study aimed to assess the impact of insomnia and depression, in combination or alone, on HRQoL measured as utility scores in a representative sample of Australian adults.

## Methods

### Study sample

The Household, Income, and Labour Dynamics in Australia (HILDA) survey is an ongoing longitudinal study that commenced in 2001. HILDA gathers comprehensive data on various aspects of family life, income, employment, and the health of over 17,000 Australians aged 15 years or above annually. The survey consists of two parts: an interview and a self-completed questionnaire (SCQ). Sleep, depression and HRQoL were collected in the SCQ part. SCQ response rates among those who completed the interview of these waves were approximately 90% [31]. A full description of sample selection and data collection was published elsewhere [31].

**Fig. 1:**
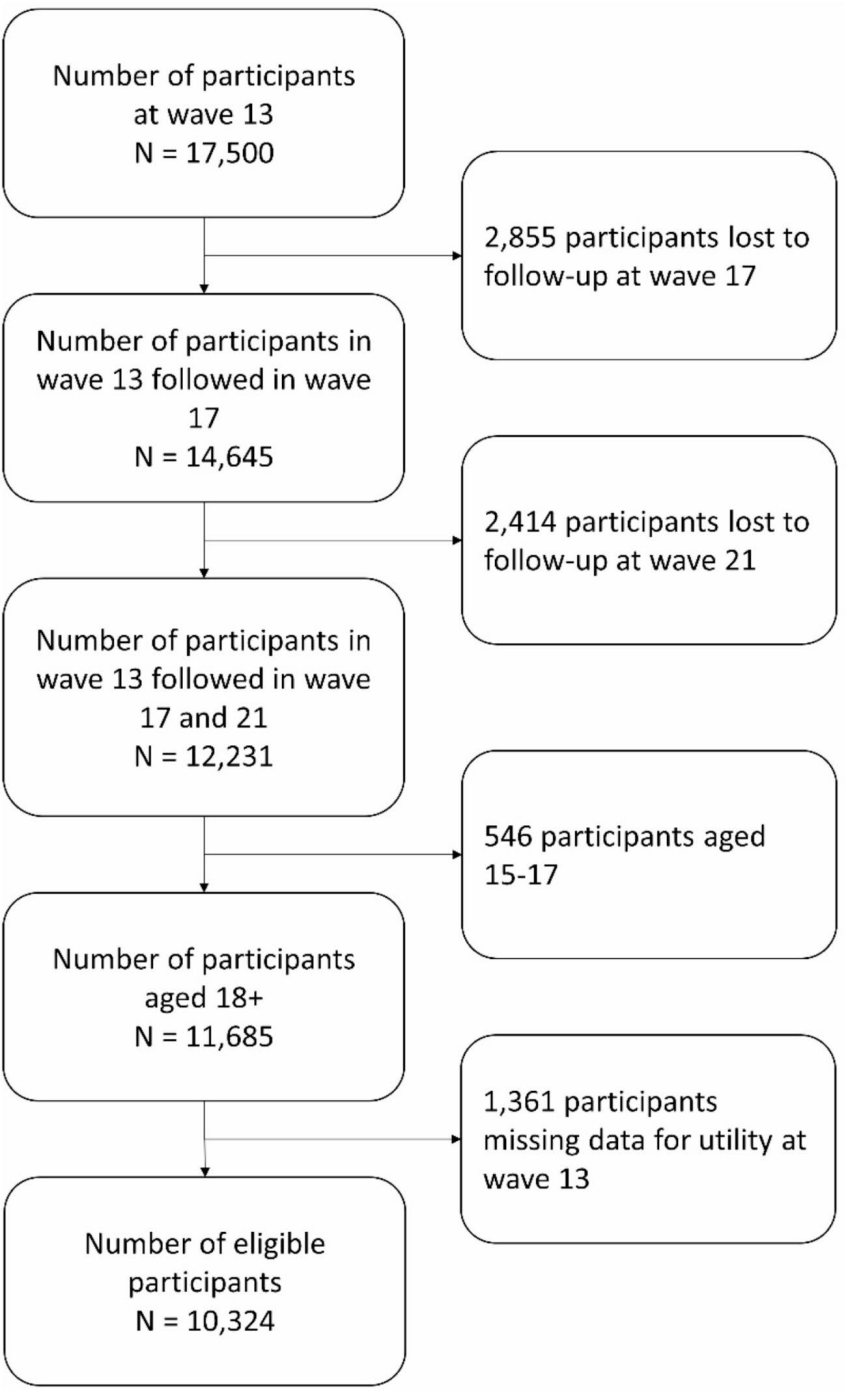
Study subject flowchart

Data from waves 13 (baseline), 17 (follow-up T1), and 21 (follow-up T2) (collected in 2013, 2017, and 2021, respectively) were used for this study since sleep items were only available in these waves [31, 32]. A total of 11,685 individuals aged 18 years or above participated in all these three waves. After excluding 1,361 participants (11.6%) due to missing data on utility scores at baseline (wave 13), the remaining 10,324 participants (30,972 observations) comprised the analytical sample (Fig. 1).

### Measures

#### Exposures

Depression was defined using a Mental Health Inventory-5 (MHI-5) [33] cut-off point of 62 or less (sensitivity: 0.74, specificity: 0.91 to define major depression and dysthymia according to DSM-III-R criteria) [34]. The MHI-5 is a brief and valid tool that contains five six-point-scale questions focusing on measuring depression and anxiety symptoms and psychological well-being (higher scores indicate better mental health) [35].

Key features of insomnia disorder according to the DSM-V are *“dissatisfaction with sleep quantity or quality with complaints of difficulty initiating or maintaining sleep”* [36]. In the HILDA survey, participants were asked to self-report how often during the past month they had trouble sleeping because of (i) difficulty getting to sleep within 30 min; (ii) wake-up in the middle of the night or early in the morning on a five-level scale. Sleep quality was also self-reported by the participants on a four-level scale. In this study, insomnia was defined as subjective complaints with sleep initiation or maintenance at least 3 times per week combined with fairly or very bad self-rated sleep quality.

#### Outcomes

Utility scores were estimated using the SF-6D, a widely used MAUI and can be derived from the SF-36 which was measured in all waves of the HILDA [22]. The validity of HILDA data for the SF-36 has been tested and published elsewhere [37]. Australian algorithm developed by Norman et al. [38] was used to value health states in this study.

#### Covariates

Potential covariates are factors previously examined that are thought to be associated with the exposure and/or outcome variables in other studies [10, 11, 29, 39–51]. These covariates for the current study comprised demographics and socio-economic status (i.e., age, sex, education, marital status, English as not first language learnt to speak as a child, employment status, regular income, and major life events), self-reported physical conditions (i.e., serious physical illnesses, obesity, and pain), behaviours and lifestyle (i.e., diet quality, physical activity, smoking, and alcohol use), and Covid-19 impact.

Definitions and values of all measures are described in Table S1 in the supplementary materials.

### Statistical analyses

We employed descriptive statistics to summarise characteristics of the analytical sample and subgroups classified based on the presence of insomnia and/or depression. Univariate tests, including t-tests of means, analysis of variance (ANOVA), and chi-square test, were used to assess the association between potential confounders and the exposures/outcomes and between insomnia and depression using data from wave 13. The association between insomnia and depression was also tested using a chi-squared test.

Data on both insomnia and depression were exclusively gathered in three waves of the HILDA survey, with a substantial four-year gap between two waves, during which no information regarding the remission and relapse of depression and insomnia was collected. Given that previous studies have highlighted the dynamic nature of depression and insomnia [52–54], a repeated measures design was employed instead of using growth models. The associations between insomnia and/or depression and utility scores were tested using linear multi-level modelling with observations nested within individuals. The null model which included random-effects only was first derived. Insomnia, depression, and the interaction between them were added to the fixed-effects model. The covariates were subsequently incorporated into the model in the following order: age and sex (Model 2), physical conditions (Model 3), the remaining variables of demographics and socio-economic status (Model 4), behaviours and lifestyle (Model 5), and Covid-19 impact (Model 6).

The final model (Model 6) was used to derive marginal mean utility scores for four groups: insomnia only, depression only, comorbid insomnia and depression, and no insomnia and depression. Tukey’s honest significant difference (HSD) was used for post-hoc pairwise comparisons to examine differences in marginal means between those with insomnia only, depression only, and comorbid insomnia and depression compared to no insomnia and depression. We also compared comorbid insomnia and depression to depression only. The pairwise differences were also expressed as standardised effect sizes using Cohen’s d (SES) with their standard errors (SE). SES was classified into small (d = 0.2), medium (d = 0.4), and large (d = 0.8) effects [55].

Residual diagnostic plots (Q-Q plots and scatter plots of residuals versus fitted values) were used to test the assumptions of normal distribution and homogeneity of variance for univariate analyses and multi-level modelling [56–58].

Statistical significance was considered when a 2-sided p-value was less than 0.05. However, for selecting confounders to be incorporated in the modelling using univariate tests, a cut-off of p-value < 0.25 was used. This is a conservative cut-off point used to minimise the risk of excluding variables that were not found to be statistically significant but might impact the modelling results [59, 60]. All analyses were performed using R version 4.3.0. The *tidyverse* package was used for descriptive statistics. For conducting multilevel modelling and calculating marginal means and effect sizes for insomnia and/or depression on utility scores, we utilised the *lme4*, *emmeans*, and *ggplot2* packages.

The mean utility scores were calculated both with and without the use of weights in the current study. If the difference between the marginal means of utility scores, with and without the utilisation of weights, was negligible, we did not apply weights for the rest of the analyses.

The missing rates for most variables were less than 7% (Refer to Table S3 in the supplementary materials); therefore, no imputations were performed. To handle missing values, pairwise deletion was applied for descriptive statistics and univariate analyses, and full information maximum likelihood (FIML) was used instead of restricted maximum likelihood (REML) for multilevel modelling.

### Sensitivity and subgroup analyses

Two other cut-off points of MHI-5 scores with different sensitivity and specificity levels were used to define depression: MHI-5 scores ≤ 54 (sensitivity: 0.63, specificity: 0.96) (Sensitivity analysis 1: SA1); and MHI-5 scores ≤ 74 (sensitivity: 0.90, specificity: 0.80) (Sensitivity analysis 2: SA2) [34].

Moreover, we used the Kessler Psychological Distress Scale (K10) to characterise depression in the subsequent analysis (Sensitivity analysis 3: SA3). The K10 is a well-validated global tool to measure psychological distress over the past four weeks in adult populations [61]. The total score was calculated as the sum of each item score, yielding a score range from 10 to 50 [62]. The choice of the cut-off point of ≥ 20 was based on validation studies and its widespread application in general practice and previous studies [62–65]. Sensitivity and specificity of K10 scores ≥ 20 to define anxiety or affective disorders were 0.66 and 0.92, respectively [63].

In line with previous studies showing a greater reduction in HRQoL associated with more severe depression, this study also investigated the effect of insomnia in people with varying depression severity levels. Given that there were no validated MHI-5 cut-off points to define depression severity, we used K10 to classify the severity of depression into three severity categories, including mild (K10 scores of 20–24), moderate (K10 scores of 25–29), and severe (K10 scores of 30–50) [63, 64] in subgroup analyses.

## Results

### Base case analysis

At baseline, the one-month prevalence of insomnia or depression was nearly 20%; and 41.3% of individuals with depression had insomnia. Table 1 summarises characteristics for the whole study sample and subgroups stratified by insomnia and depression: no insomnia or depression, insomnia only, depression only, and comorbid insomnia and depression. At baseline (wave 13), approximately 11% of individuals had insomnia only, and a similar proportion had depression only, while 8.2% had both insomnia and depression. The proportions for depression only and comorbid insomnia and depression at T2 were slightly higher, at 15% and 11%, respectively. The mean age of the sample at the baseline was 45.7 ± 16.5, with females accounting for 54.6%.

**Table 1:** Characteristics of the study subjects by subgroups of depression and insomnia in the base case analysis

Variables		Wave 13, *N* (%)					Wave 17, *N* (%)					Wave 21, *N* (%)				
		ALL	NONE	INS	DEP	BOTH	ALL	NONE	INS	DEP	BOTH	ALL	NONE	INS	DEP	BOTH
N (%)		10,324 (100.0)	6,998 (68.8)	1,151 (11.3)	1,184 (11.6)	833 (8.2)	10,324 (100.0)	6,537 (66.7)	1,170 (11.9)	1,206 (12.3)	887 (9.1)	10,324 (100.0)	5,987 (61.9)	1,156 (12.0)	1,475 (15.3)	1,054 (10.9)
Utility scores Mean (SD)		0.662 (0.236)	0.740 (0.174)	0.642 (0.210)	0.458 (0.228)	0.338 (0.256)	0.641 (0.246)	0.729 (0.182)	0.612 (0.216)	0.445 (0.235)	0.309 (0.256)	0.612 (0.253)	0.711 (0.186)	0.613 (0.212)	0.441 (0.237)	0.299 (0.248)
Age Mean (SD)		45.7 (16.5)	46.1 (16.8)	47.1 (15.8)	42.7 (16.0)	44.8 (15.2)	49.7 (16.5)	50.5 (16.6)	51.4 (15.7)	46.5 (16.4)	47.1 (14.9)	53.7 (16.5)	55.1 (16.4)	54.8 (15.6)	50.1 (16.4)	50.9 (15.3)
Sex	Male	4,691 (45.4)	3,354 (47.9)	451 (39.2)	512 (43.2)	306 (36.7)	4,691 (45.4)	3,113 (47.6)	463 (39.6)	538 (44.6)	335 (37.8)	4,691 (45.4)	2,870 (47.9)	472 (40.8)	636 (43.1)	391 (37.1)
	Female	5,633 (54.6)	36,44 (52.1)	700 (60.8)	672 (56.8)	527 (63.3)	5,633 (54.6)	3,424 (52.4)	707 (60.4)	668 (55.4)	552 (62.2)	5,633 (54.6)	3,117 (52.1)	684 (59.2)	839 (56.9)	663 (62.9)
Education level	High	2,947 (28.6)	2,168 (31.0)	291 (25.3)	294 (24.8)	170 (20.4)	3,280 (31.8)	2,250 (34.4)	337 (28.8)	376 (31.2)	189 (21.3)	3,416 (33.1)	2,130 (35.6)	354 (30.6)	478 (32.4)	281 (26.7)
	Middle	5,027 (48.7)	3,394 (48.5)	566 (49.2)	605 (51.1)	397 (47.7)	4,877 (47.3)	3,013 (46.1)	573 (49.0)	568 (47.1)	454 (51.2)	4,810 (46.6)	2,738 (45.8)	561 (48.5)	694 (47.1)	510 (48.4)
	Low	2,345 (22.7)	1,432 (20.5)	293 (25.5)	285 (24.1)	266 (31.9)	2,162 (21.0)	1,270 (19.4)	259 (22.2)	262 (21.7)	244 (27.5)	2,093 (20.3)	1,114 (18.6)	241 (20.8)	303 (20.5)	263 (25.0)
Marital status	Partnered	7,210 (69.8)	5,095 (72.8)	813 (70.6)	705 (59.5)	494 (59.3)	7,340 (71.1)	4,835 (74.0)	869 (74.3)	752 (62.4)	552 (62.2)	7,294 (70.7)	4,465 (74.6)	853 (73.9)	969 (65.7)	630 (59.8)
	Non-partnered	3,114 (30.2)	1,903 (27.2)	338 (29.4)	479 (40.5)	339 (40.7)	2,983 (28.9)	1,701 (26.0)	301 (25.7)	454 (37.6)	335 (37.8)	3,027 (29.3)	1,521 (25.4)	302 (26.1)	506 (34.3)	423 (40.2)
English as 2nd language	Yes	9,279 (89.9)	6,280 (89.8)	1,066 (92.6)	1,037 (87.6)	758 (91.0)	9,279 (89.9)	5,869 (89.8)	1,086 (92.8)	1051 (87.2)	823 (92.8)	9,279 (89.9)	5,357 (89.5)	5,357 (89.5)	1,307 (88.6)	970 (92.0)
	No	1,044 (10.1)	717 (10.2)	85 (7.4)	147 (12.4)	75 (9.0)	1,044 (10.1)	668 (10.2)	84 (7.2)	154 (12.8)	64 (7.2)	1,044 (10.1)	630 (10.5)	630 (10.5)	168 (11.4)	84 (8.0)
Employment status	Employed	6,974 (67.6)	4956 (70.8)	755 (65.6)	736 (62.2)	444 (53.3)	6,769 (65.6)	4,477 (68.5)	733 (62.6)	770 (63.8)	471 (53.1)	6,319 (61.2)	3,766 (62.9)	684 (59.2)	888 (60.2)	592 (56.2)
	Unemployed/ Not in the labour force	3,350 (32.4)	2042 (29.2)	396 (34.4)	448 (37.8)	389 (46.7)	3,555 (34.4)	2,060 (31.5)	437 (37.4)	436 (36.2)	416 (46.9)	4,005 (38.8)	2,221 (37.1)	472 (40.8)	587 (39.8)	462 (43.8)
Household income Mean (SD) (per A$10,000)		11.6 (10.2)	12.2 (10.5)	11.4 (10.6)	10.3 (9.3)	9.5 (8.1)	12.4 (12.6)	13.2 (13.6)	12.0 (11.7)	11.1 (10.6)	10.2 (9.9)	13.5 (12.6)	14.2 (13.6)	13.0 (10.5)	12.4 (9.2)	11.9 (11.1)
Social support	Medium-High	6,721 (66.2)	5,270 (76.3)	751 (66.5)	396 (34.1)	219 (26.7)	6,448 (65.9)	4,959 (77.4)	775 (67.2)	393 (33.5)	225 (26.0)	6,048 (62.3)	4,412 (74.4)	725 (63.4)	534 (36.8)	318 (30.4)
	Low	3,436 (33.8)	1,633 (23.7)	378 (33.5)	764 (65.9)	602 (73.3)	3,341 (34.1)	1,451 (22.6)	378 (32.8)	780 (66.5)	640 (74.0)	3,660 (37.7)	1,517 (25.6)	418 (36.6)	919 (63.2)	728 (69.6)
Major life events	No	3,958 (39.4)	2,848 (41.6)	404 (36.0)	416 (36.3)	239 (29.8)	4,141 (42.4)	2,898 (45.2)	445 (39.0)	417 (35.6)	285 (33.1)	4,341 (44.8)	2,823 (47.6)	503 (44.0)	594 (41.0)	352 (34.2)
	Yes	6,100 (60.6)	3,995 (58.4)	718 (64.0)	730 (63.7)	564 (70.2)	5,637 (57.6)	3,510 (54.8)	696 (61.0)	755 (64.4)	576 (66.9)	5,359 (55.2)	3,112 (52.4)	639 (56.0)	856 (59.0)	676 (65.8)
Sleep medication	No	9,400 (91.6)	6,681 (95.8)	954 (83.0)	1,045 (88.7)	606 (73.1)	9,045 (91.0)	6,208 (95.3)	980 (84.3)	1,049 (87.6)	652 (74.0)	8,663 (88.8)	5,623 (94.2)	929 (80.4)	1,242 (85.1)	768 (73.0)
	Yes	867 (8.4)	294 (4.2)	196 (17.0)	133 (11.3)	223 (26.9)	897 (9.0)	303 (4.7)	183 (15.7)	149 (12.4)	229 (26.0)	1,094 (11.2)	344 (5.8)	227 (19.6)	218 (14.9)	284 (27.0)
Depression medication	No	9,811 (95.0)	6,840 (97.7)	1,106 (96.1)	1,052 (88.9)	667 (80.1)	9,525 (92.3)	6,320 (96.7)	1,106 (94.5)	994 (82.4)	644 (72.6)	9,317 (90.2)	5,731 (95.7)	1,080 (93.4)	1,181 (80.1)	756 (71.7)
	Yes	513 (5.0)	158 (2.3)	45 (3.9)	132 (11.1)	166 (19.9)	799 (7.7)	217 (3.3)	64 (5.5)	212 (17.6)	243 (27.4)	1,007 (9.8)	256 (4.3)	76 (6.6)	294 (19.9)	298 (28.3)
Chronic pain	No	9,527 (92.3)	6,663 (95.2)	1,018 (88.4)	1,062 (89.7)	646 (77.6)	9,326 (90.3)	6,155 (94.2)	996 (85.1)	1,052 (87.2)	663 (74.7)	9,197 (89.1)	5,575 (93.1)	997 (86.2)	1,270 (86.1)	790 (75.0)
	Yes	797 (7.7)	335 (4.8)	133 (11.6)	122 (10.3)	187 (22.4)	998 (9.7)	382 (5.8)	174 (14.9)	154 (12.8)	224 (25.3)	1,127 (10.9)	412 (6.9)	159 (13.8)	205 (13.9)	264 (25.0)
Serious physical illnesses	No	6,253 (60.6)	4,449 (63.6)	609 (52.9)	700 (59.1)	406 (48.7)	5,845 (56.6)	3,891 (59.5)	556 (47.5)	685 (56.8)	407 (45.9)	5,275 (51.1)	3,192 (53.3)	511 (44.2)	788 (53.4)	427 (40.5)
	Yes	4,071 (39.4)	2,549 (36.4)	542 (47.1)	484 (40.9)	427 (51.3)	4,479 (43.4)	2,646 (40.5)	614 (52.5)	521 (43.2)	480 (54.1)	5,049 (48.9)	2,795 (46.7)	645 (55.8)	687 (46.6)	627 (59.5)
Obesity	No	7,540 (75.3)	5,297 (77.9)	795 (70.5)	838 (73.6)	507 (63.5)	7,027 (72.3)	4,765 (74.9)	786 (68.7)	820 (70.8)	500 (59.4)	6,688 (69.0)	4,293 (72.7)	746 (65.2)	964 (66.6)	580 (56.0)
	Yes	2,474 (24.7)	1,507 (22.1)	332 (29.5)	300 (26.4)	291 (36.5)	2,698 (27.7)	1,600 (25.1)	358 (31.3)	339 (29.2)	342 (40.6)	3,010 (31.0)	1,610 (27.3)	398 (34.8)	483 (33.4)	456 (44.0)
Diet quality	Good	3,267 (34.1)	2,408 (37.0)	351 (32.3)	273 (24.9)	199 (25.9)	2,958 (32)	2,066 (34.5)	360 (32.6)	276 (24.7)	197 (23.6)	2,501 (27.7)	1,681 (30.7)	289 (27.0)	308 (22.9)	184 (18.6)
	Poor	6,319 (65.9)	4,101 (63.0)	737 (67.7)	823 (75.1)	568 (74.1)	6,285 (68)	3,928 (65.5)	743 (67.4)	841 (75.3)	639 (76.4)	6,523 (72.3)	3,803 (69.3)	783 (73.0)	1,035 (77.1)	804 (81.4)
Physical activity	High frequency	3,617 (35.1)	2,709 (38.8)	349 (30.4)	319 (27.0)	181 (21.8)	3,231 (32.5)	2,374 (36.4)	342 (29.3)	307 (25.5)	160 (18.1)	3,345 (34)	2,344 (39.2)	355 (30.7)	389 (26.4)	208 (19.7)
	Low frequency	6,688 (64.9)	4,280 (61.2)	798 (69.6)	861 (73.0)	650 (78.2)	6,722 (67.5)	4,156 (63.6)	825 (70.7)	897 (74.5)	726 (81.9)	6,506 (66)	3,638 (60.8)	800 (69.3)	1,086 (73.6)	846 (80.3)
Alcohol use	Low risk	8,736 (85.1)	5,896 (84.8)	954 (83.1)	,1032 (87.6)	721 (87.1)	8,381 (84.6)	5,435 (83.7)	974 (83.7)	1,057 (88.2)	771 (87.3)	8,201 (83.4)	4,918 (82.3)	955 (82.8)	1,266 (86.1)	904 (85.8)
	High risk	1,526 (14.9)	1,056 (15.2)	194 (16.9)	146 (12.4)	107 (12.9)	1,527 (15.4)	1,062 (16.3)	190 (16.3)	141 (11.8)	112 (12.7)	1,636 (16.6)	1,059 (17.7)	198 (17.2)	205 (13.9)	149 (14.2)
Smoking	Never/Former smoker	8,567 (83.3)	6,027 (86.4)	923 (80.5)	923 (78.3)	582 (70.1)	8,470 (85.3)	5,753 (88.4)	965 (82.8)	964 (80.3)	657 (74.3)	85,55 (87.1)	53,30 (89.3)	1,002 (86.9)	1,236 (83.9)	837 (79.7)
	Smoker	1,717 (16.7)	945 (13.6)	224 (19.5)	256 (21.7)	248 (29.9)	1,454 (14.7)	757 (11.6)	201 (17.2)	237 (19.7)	227 (25.7)	12,71 (12.9)	637 (10.7)	151 (13.1)	238 (16.1)	213 (20.3)
Life changed by Covid	Not impacted											955 (27.6)	621 (8.9)	102 (8.9)	121 (10.2)	86 (10.3)
	Impacted with a great extent											2,505 (72.4)	1,614 (23.1)	301 (26.2)	307 (26.0)	246 (29.5)
	Impacted with a moderate extent											3,651 (51.3)	2,527 (36.1)	394 (34.3)	414 (35.0)	260 (31.2)
	Impacted with a great extent											3,207 (34.3)	2,234 (31.9)	353 (30.7)	340 (28.8)	241 (28.9)

NONE: No insomnia and depression, INS: Insomnia, DEP: Depression, BOTH: Comorbid insomnia and depression

Missing rates for all variables were presented Table S3 in the supplementary material

The mean utility scores, whether weighted or unweighted, were nearly equal, as presented in Table S2 in the supplementary materials. The weighted and unweighted mean utility scores of the study sample were 0.636 ± 0.247 and 0.639 ± 0.246, respectively.

Table 2 presents the results of multilevel modelling for the base case analysis. The intraclass correlation coefficient (ICC) for the null model was 0.599. As shown in Table 2, insomnia, depression, and the interaction term between insomnia and depression presented significant associations with the decrement of utility scores in both unadjusted (Model 1) and adjusted models (Model 2–6). In model 6, which included all covariates, the main effect showed that insomnia or depression independently decreased utility scores by 0.058 (95% CI: 0.064 to 0.051) and 0.210 (95% CI: 0.216 to 0.203), respectively. The interaction term between insomnia and depression suggested that the combined effect of insomnia and depression on utility scores was synergistic, meaning comorbid insomnia and depression led to an additional decrease of 0.023 (95% CI: -0.034 to -0.013) in utility score beyond the sum of their individual effects alone.

**Table 2:** Association of Insomnia and depression with utility scores (β, 95% CI)

	Model 1	Model 2	Model 3	Model 4	Model 5	Model 6
**Fixed effects**						
Intercept	0.709 (0.706–0.713)^***^	0.835 (0.828–0.843)^***^	0.837 (0.830–0.844)^***^	0.865 (0.856–0.874)^***^	0.903 (0.893–0.913)^***^	0.902 (0.888–0.916)^***^
Insomnia	-0.078 (-0.084 – -0.071)^***^	-0.077 (-0.083 – -0.070)^***^	-0.066 (-0.073 – -0.060)^***^	-0.061 (-0.067 – -0.055)^***^	-0.058 (-0.064 – -0.051)^***^	-0.058 (-0.064 – -0.051)^***^
Depression	-0.231 (-0.237 – -0.225)^***^	-0.238 (-0.244 – -0.231)^***^	-0.233 (-0.239 – -0.227)^***^	-0.213 (-0.219 – -0.207)^***^	-0.21 (-0.217 – -0.204)^***^	-0.210 (-0.216 – -0.203)^***^
Insomnia*depression	-0.029 (-0.040 – -0.018)^***^	-0.029 (-0.040 – -0.019)^***^	-0.022 (-0.033 – -0.012)^***^	-0.023 (-0.033 – -0.013)^***^	-0.024 (-0.034 – -0.013)^***^	-0.023 (-0.034 – -0.013)^***^
Age (centring at 18)		-0.003 (-0.004 – -0.003)^***^	-0.002 (-0.002 – -0.002)^***^	-0.002 (-0.002 – -0.001)^***^	-0.002 (-0.002 – -0.002)^***^	-0.002 (-0.002 – -0.002)^***^
Sex (Female)		-0.031 (-0.037 – -0.025)^***^	-0.024 (-0.029 – -0.019)^***^	-0.024 (-0.029 – -0.018)^***^	-0.022 (-0.027 – -0.016)^***^	-0.021 (-0.027 – -0.016)^***^
Serious physical illnesses			-0.051 (-0.056 – -0.046)^***^	-0.045 (-0.050 – -0.040)^***^	-0.045 (-0.050 – -0.040)^***^	-0.045 (-0.050 – -0.040)^***^
Obesity			-0.041 (-0.046 – -0.035)^***^	-0.037 (-0.042 – -0.032)^***^	-0.032 (-0.037 – -0.027)^***^	-0.032 (-0.037 – -0.027)^***^
Chronic pain			-0.177 (-0.184 – -0.170)^***^	-0.172 (-0.180 – -0.165)^***^	-0.169 (-0.177 – -0.162)^***^	-0.169 (-0.177 – -0.162)^***^
Education (Medium)				-0.01 (-0.016 – -0.004)^***^	-0.007 (-0.013 – -0.001)^*^	-0.008 (-0.014 – -0.002)^*^
Education (Low)				-0.015 (-0.022 – -0.007)^***^	-0.006 (-0.014–0.002)	-0.007 (-0.014–0.001)
Non-partnered				-0.011 (-0.016 – -0.005)^***^	-0.010 (-0.016 – -0.005)^***^	-0.010 (-0.015 – -0.005)^***^
English as 2nd language				-0.012 (-0.021 – -0.003)^*^	-0.006 (-0.015–0.003)	-0.005 (-0.014–0.004)
Unemployed or not in the labour force				-0.038 (-0.043 – -0.033)^***^	-0.04 (-0.046 – -0.035)^***^	-0.04 (-0.046 – -0.035)^***^
Income (A$10,000)				0.001 (0.000–0.001)^***^	0.000 (0.000–0.001)^***^	0.000 (0.000–0.001)^***^
Low social support				-0.052 (-0.057 – -0.047)^***^	-0.050 (-0.055 – -0.045)^***^	-0.050 (-0.055 – -0.045)^***^
Major life events				-0.024 (-0.028 – -0.020)^***^	-0.024 (-0.028 – -0.020)^***^	-0.024 (-0.028 – -0.020)^***^
Low diet quality					-0.012 (-0.017 – -0.007)^***^	-0.012 (-0.017 – -0.007)^***^
Low frequency of physical activity					-0.045 (-0.049 – -0.040)^***^	-0.045 (-0.049 – -0.040)^***^
Smoking					-0.017 (-0.024 – -0.010)^***^	-0.017 (-0.024 – -0.010)^***^
High-risk alcohol use					0.013 (0.007–0.020)^***^	0.013 (0.007–0.020)^***^
Life changed by Covid (Great extent)						-0.009 (-0.020–0.001)
Life changed by Covid (Moderate extent)						0.004 (-0.006–0.014)
Life changed by Covid (Little extent)						0.004 (-0.006–0.014)
**Random effect**						
Level-2 variance	0.020	0.020	0.019	0.019	0.019	0.019
Level-1 variance	0.021	0.018	0.012	0.010	0.010	0.010
**Summary**						
No. participants	10,315	10,315	10,281	10,228	10,043	10,038
No. observations	29,163	29,163	28,462	27,549	25,399	25,390
Marginal R-squared	0.246	0.307	0.418	0.455	0.471	0.472
Conditional R-squared	0.630	0.634	0.638	0.648	0.654	0.655

*P* < 0.05, ^**^P < 0.01, ^***^P < 0.001

**Fig. 2:**
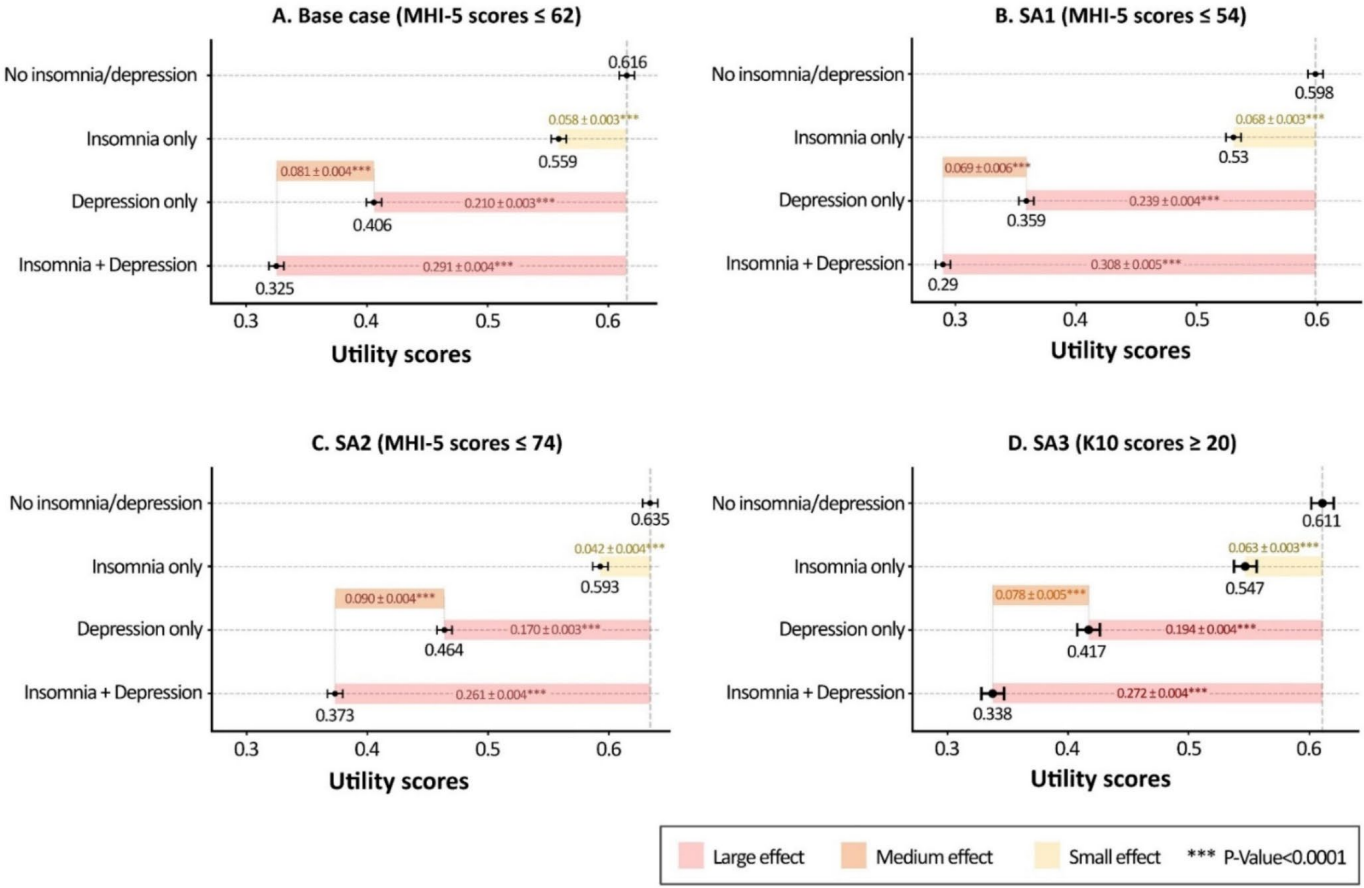
Marginal mean utility scores and standard error of depression and/or insomnia. Adjusted for age, sex, mental and physical health, demographics, lifestyle, and Covid impact. (The error bars present marginal mean utility scores and their standard errors for each subgroup defined by insomnia and depression. The figures in coloured bars are marginal mean differences in utility scores between the two subgroups, along with their standard errors: insomnia only, depression only, and comorbid insomnia and depression compared to no insomnia and depression, and comorbid insomnia and depression compared to depression only)

After adjusting for all covariates and the interaction effect of insomnia and depression, marginal mean utility scores for individuals with insomnia only, depression only, and comorbid insomnia and depression were calculated to be 0.559 (95% CI: 0.550–0.568), 0.406 (95% CI: 0.398–0.415) and 0.325 (95% CI: 0.316–0.335), respectively. These scores were significantly lower than those without insomnia and depression (0.616, 95% CI: 0.609–0.624). Figure 2 and Table S6 in the supplementary materials present differences in marginal mean utility scores and effect sizes across the pairwise comparisons. The standardised effect sizes for insomnia only, depression only and comorbid insomnia and depression were 0.420 (small), 1.530 (large), and 2.120 (large), respectively. For individuals with depression, the marginal mean utility score of those experiencing insomnia was 0.081 (SE:0.004, t-ratio: 18.157, *P* < 0.0001) lower than those not experiencing it (SES: 0.591, moderate effect).

### Sensitivity and subgroup analyses

A comparison between the prevalence of depression and insomnia as well as mean utility scores in base case and sensitivity analyses is shown in Table SS in the supplementary materials. The prevalence values and the mean utility scores for subgroups using a cut-off point of K10 scores ≥ 20 to define depression (SA3) were nearly the same as the base case analysis.

All sensitivity analyses indicated that both insomnia and depression were significantly associated with the decreased utility scores (Table S5 in the supplementary materials). The regression coefficients of insomnia, depression, and the interaction term between the base case and SA3 were nearly similar. For SA1, the decrement of utility scores associated with insomnia or depression was slightly larger than the base case analysis, and the interaction term was not statistically significant (β: -0.001, 95% CI: -0.013 to 0.011). For SA2, while the coefficient for either insomnia or depression was slightly smaller, the coefficient for the interaction effect doubled compared to the base case analysis (β: -0.005, 95% CI: -0.060 to -0.039). Marginal mean utility scores of all subgroups and their pairwise differences, along with effect sizes, derived from all SAs are shown in Fig. 2B-D and Table S6 in the supplementary materials.

**Fig. 3:**
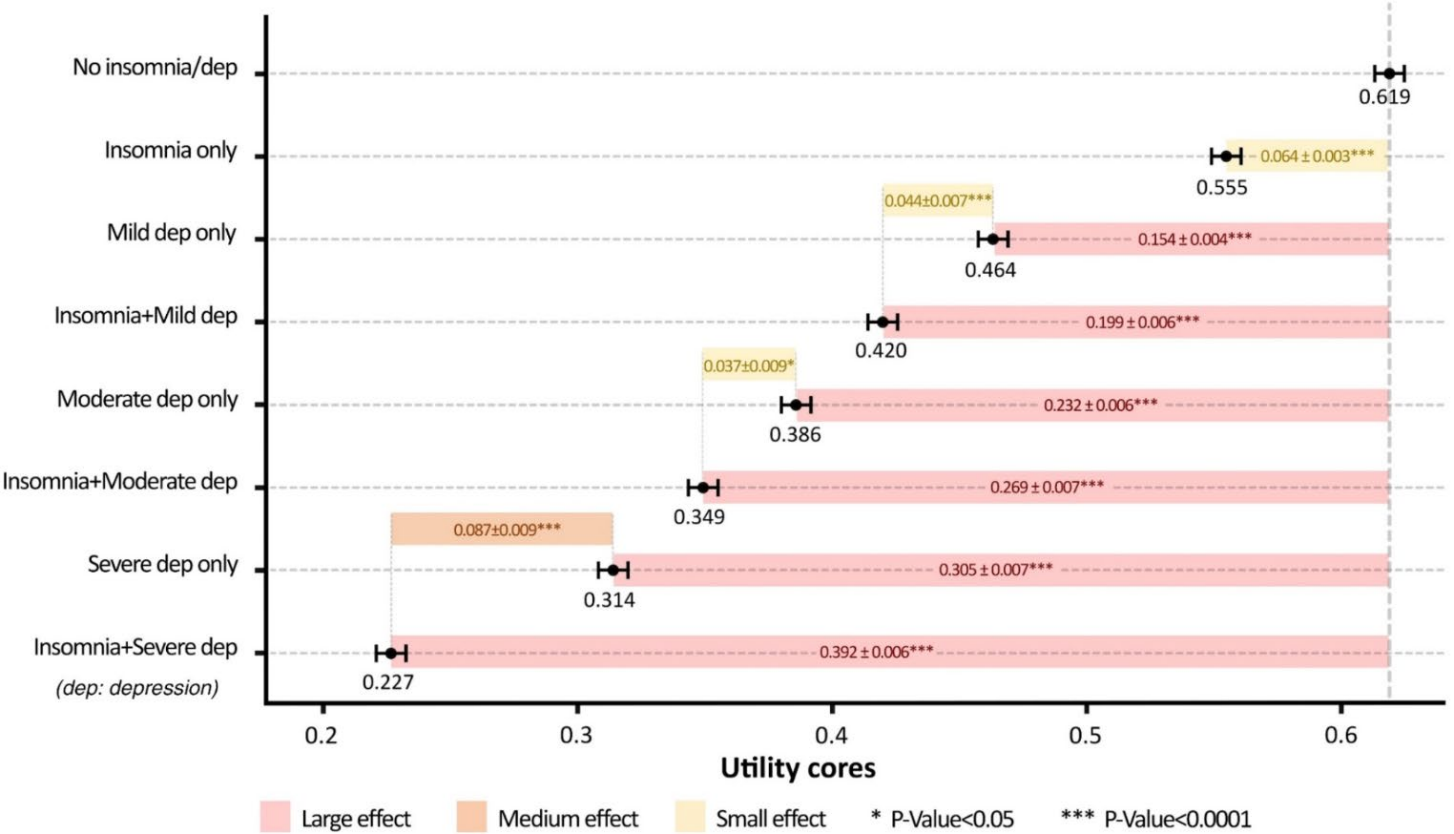
Marginal mean utility scores and standard error of different depression severity with/without insomnia. Adjusted by age, sex, mental and physical health, demographics, lifestyle, and Covid impact. (The error bars present marginal mean utility scores and their standard errors for each subgroup defined by insomnia and depression. The figures in coloured bars are marginal mean differences in utility scores between the two subgroups, along with their standard errors: insomnia only, depression only, and comorbid insomnia and depression compared to no insomnia and depression, and comorbid insomnia and depression compared to depression only.)

The subgroup analyses using K10 cut-off points found that higher levels of depression severity led to greater utility decrements. This is demonstrated by the regression coefficients from the multilevel model (Table S7 in the supplementary materials), as well as marginal mean utility scores for each subgroup defined by insomnia and depression (Fig. 3). In cases of mild/moderate depression, the interaction effect between insomnia and depression expressed the overlapping effect of insomnia and depression on utility scores (β: 0.019, 95% CI: 0.005 to 0.033 for mild; β: 0.027, 95% CI: 0.009 to 0.045 for moderate). However, for severe depression, the interaction effect indicated an additional decrement of utility scores attributed to the co-occurrence of insomnia and depression (β: -0.023, 95% CI: -0.042 to -0.005). Figure 3 and Table S8 in the supplementary materials present marginal mean utility scores for each subgroup defined by insomnia and depression and the mean differences in utility scores with effect sizes of insomnia and/or depression relative to no insomnia and depression and of comorbid insomnia and depression relative to depression only. Marginal mean scores were lower when depression severity was more severe. For people with mild depression, the marginal mean score was 0.464 ± 0.005 for those without insomnia and 0.420 ± 0.006 for those with insomnia. For people with moderate depression, the marginal mean score was 0.386 ± 0.007 for those without insomnia and 0.349 ± 0.007 for those with insomnia. For people with severe depression, the marginal mean score was 0.314 ± 0.008 for those without insomnia and 0.227 ± 0.007 for those with insomnia. The effect size of insomnia impact on utility scores was small for individuals with mild/moderate depression (SES: 0.322 for mild depression and 0.267 for moderate depression) but medium for individuals with severe depression (SES: 0.631).

## Discussion

Our study found that insomnia and depression had substantive impacts on HRQoL as measured by SF-6D utility scores. The impairment of utility scores associated with comorbid insomnia and depression was, overall, significantly larger than the sum of their individual effects. To the best of our knowledge, this is the first study that has examined the reduction in utility scores associated with insomnia and depression, with the explicit consideration of the impacts of their interaction.

The subgroup analysis showed that increasing depression severity was associated with decreased levels of utility scores. The results of sensitivity analyses were also in line with this. The difference in utility scores between individuals with and without depression (regardless of the presence of insomnia) was found to be higher when using MHI-5 ≤ 54 (SA1) to define depression than when using MHI-5 ≤ 62 (base case) or MHI-5 ≤ 74 (SA2). This may be due to MHI-5 ≤ 54 capturing individuals with more severe depression. Previous studies have also observed similar findings [28, 66–68]. Moreover, depression severity also affected the combined effect between insomnia and depression on HRQoL. For mild-to-moderate depression, the interaction term in the model suggested the effects of insomnia and depression overlapped. For severe depression, the comorbidity of insomnia and depression incurred an additional negative effect greater than the overlapping effect. Further studies are required to strengthen this result.

Our study has revealed the burden of insomnia in those with depression. We observed a high prevalence of insomnia in this group (32–46%), which aligns with previous epidemiological studies [3, 69]. Insomnia significantly decreased HRQoL after adjusting for the effect of depression and other covariates. These findings are in line with another study that observed significant associations between higher severity levels of insomnia and lower utility scores in American adults with major depressive disorder [70]. Our study findings also underscored the bidirectional associations between insomnia and depression found in prior research [4–6]. The impairment of HRQoL associated with insomnia in adults with depression was found to have a medium effect size, while insomnia resulted in a small effect size on the reduction of HRQoL in those without depression. However, in practice, little effort is made to directly address insomnia when insomnia and depression concurrently occur [2, 71–73], and management of depression is expected to address both depression and insomnia symptoms [2, 71]. Indeed, chronic insomnia is 1.8 to 3.5 times more likely to perpetuate depression [8], and residual insomnia symptoms may increase the risk of depression relapse [74]. Current studies have shown that adding interventions targeting insomnia (including cognitive behavioural therapy and sedative-hypnotics) to depression treatments results in better HRQoL and more QALYs gained compared to depression treatment alone [75–77]. Our study findings suggest targeting the management of insomnia may improve the HRQoL of individuals with depression.

Comparing the utility scores measured in this study with previous research is limited. We were unable to compare the utility scores in the current study to population norms because there have been no publications on Australian population norms for the SF-6D. Moreover, the variety of MAUIs and methods applied to value health states also makes comparisons across studies difficult [78]. In this study, the utility scores were derived from the Australian algorithm developed by Norman et al. [38] who used the methods of discrete choice experiments (DCE) for the valuation of SF-6D health states. Other studies that have used the SF-6D have used the algorithm published by J. Brazier et al. [22] that used standard gamble (SG) instead [29, 66, 70]. The mean utility scores of individuals with insomnia or depression estimated in this current study were generally lower than the other studies. Norman et al. [38] explained that whereas J. Brazier et al. [22] valued no health states measured by the SF-6D as being below zero, there were 5% of health states in the Australian valuation study that were assigned a negative value.

### Strengths and limitations

Our study estimated utility scores associated with insomnia and/or depression using data from a national survey with a large study sample size of 10,324 adults and an Australian algorithm [38]. Apart from showing that depression and insomnia are associated with substantive losses in utility, the utility scores derived from this study can be used in future clinical and economic studies on insomnia and depression. However, since we only used the Australian algorithm to value SF-6D health states, the generalisability of utility values estimated in this study to other countries is unclear. The preference-based scoring algorithm of the Australian population may not reflect the preferences of people in other countries. Although this study utilised validated cut-off points of MHI-5 or K10 [34, 63, 79, 80], the absence of formal diagnostic questions of depression is a study limitation. The measure of insomnia did not include the DSM-V diagnostic duration criteria, which require sleep difficulties to last for at least three months. As a result, our study findings are likely to be conservative since insomnia may also include cases of acute and short-term sleep disturbances. Nevertheless, the estimated prevalence was consistent with other large epidemiological studies [3, 69]. The application of multi-level mixed-effect models with three-wave panel data nested within individuals might offer more insights when working with a dataset containing a larger number of observations. This approach also allowed us to control individual time-constant unobserved factors as well as time effects and dynamics [81]. However, the repeated measures design with lengthy intervals between the HILDA waves restricted causal inferences between insomnia/depression and decreased utility scores. Finally, the impact of depression severity on utility scores was analysed in this study, but the effect of different insomnia severity levels on the outcomes was not examined.

## Conclusions

This study has provided new insights into the burden associated with insomnia and depression in adult populations. The impact of insomnia and depression on utility scores, either individually or in combination, was significant. The co-occurrence of insomnia and depression was associated with an additional reduction in utility scores beyond the sum of their individual effects. Alleviating insomnia symptoms is suggested to be important for improving the HRQoL of adults with depression. The utility scores estimated in this study can be utilised in future clinical or economic studies.

## Electronic supplementary material

Below is the link to the electronic supplementary material.

Supplementary Material 1

## Data Availability

The HILDA unit data can be accessed upon request via the Dataverse platform (https://dataverse.ada.edu.au/dataverse.xhtml?alias=hilda).
