# Supplementary material for: Quality of life impacts associated with comorbid insomnia and depression in adult population
Source: Qual Life Res. 2024 Sep 26;33(12):3283–98. doi: 10.1007/s11136-024-03793-y (PMC11599622; doi:10.1007/s11136-024-03793-y)
Supplement: Supplementary file 1 — Supplementary Material 1 [file 11136_2024_3793_MOESM1_ESM.docx]

**SUPPLEMENTARY MATERIAL**

**Quality of life impacts associated with comorbid insomnia and depression in adult population**

***Quality of Life Research***

Phuong Hong Le, MHE^1*^, Long Khanh-Dao Le, PhD^1^, Shantha M.W. Rajaratnam, PhD^2^,

Cathrine Mihalopoulos, PhD^1^

^1^Monash University Health Economics Group (MUHEG), School of Public Health and Preventative Medicine, Monash University, Melbourne, Australia

^2^School of Psychological Sciences and Turner Institute for Brain and Mental Health, Monash University, Clayton, Australia

***Corresponding author:**

Phuong H. Le, Monash University Health Economics Group, School of Public Health and Preventative Medicine, Monash University, Level 4, 553 St Kilda Road, Melbourne VIC 3004.

Email address: [Ph.le@monash.edu](mailto:Ph.le@monash.edu)

**Content**

[Table S1. Description of variables included in analyses 2](#_Toc176973332)

[Table S2. Unweighted versus weighted mean of utility scores 5](#_Toc176973333)

[Table S3. Missing values in the analytical sample 6](#_Toc176973334)

[Table S4. Prevalence and utility scores across all definitions of depression at baseline (Wave 13) 7](#_Toc176973335)

[Table S5. Multi-level modelling results with different definitions of depression 8](#_Toc176973336)

[Table S6. Marginal mean differences in utility scores and effect size Cohen’s d of pairwise comparisons between subgroups defined by insomnia and depression 9](#_Toc176973337)

[Table S7. Multi-level modelling results with depression severity defined by K10 10](#_Toc176973338)

[Table S8. Marginal mean differences in utility scores and effect size Cohen’s d of pairwise comparisons between subgroups defined by insomnia and depression severity levels 11](#_Toc176973339)

# Table S1. Description of variables included in analyses

| **Variable** | **Description** |
| --- | --- |
| Utility scores | Continuous (range: 0-1, sometimes lower than 0). Derived using Australian algorithm[1] for the SF-6D. |
| Insomnia | Binary (yes/no). During the past month, difficulties initiating or maintaining sleep at least three nights per week (based on a five-point scale) and fairly bad or worse sleep quality (based on a four-point scale). |
| Depression | Binary (yes/no).   - Base case analysis: depression defined by an MHI-5 cut-off point of ≤ 62 [2]; - Sensitivity analysis 1 (SA1): depression defined by an MHI-5 cut-off point of ≤ 54 [2]; - Sensitivity analysis 2 (SA2): depression defined by an MHI-5 cut-off point of ≤ 74 [2]. - Sensitivity analysis 3 (SA3): depression defined by an K10 cut-off point of ≥ 20 [3]. |
| Depression severity | Categorical (no/mild/moderate/severe). Defined based on K10 scores [3].   - No: < 20 scores - Mild: 20-24 scores - Moderate: 25-29 scores - Severe: 30-50 scores |
| Age | Continuous (18 or above). Age at last birthday as of 30 June for that wave. |
| Sex | Binary (male/female). Self-reported sex. |
| Serious physical illnesses | Binary (yes/no). Self-reported diagnosis of at least one of the following illnesses: arthritis or osteoporosis, asthma, cancer, chronic bronchitis or emphysema, Type 1 diabetes, Type 2 diabetes, heart disease, high blood pressure or hypertension, any other serious circulatory condition (e.g., stroke, hardening of the arteries). |
| Obesity | Binary (yes/no). BMI ≥ 30 kg/m^2^. BMI was measured by self-reported weight in kilograms divided by self-reported height in meters squared. |
| Chronic pain | Binary (yes/no). Self-reported long-term pain. |
| Education | Categorical (high/medium/low). Self-reported highest education level.   - High: masters or doctorate, graduate diploma, graduate certificate, or bachelor or honours - Intermediate: advanced diploma, diploma, Certificate III or IV, or completed high school - Low: not completed high school |
| Partnered | Binary (partnered/non-partnered). Being in a marital relationship.   - Partnered: married or de facto relationship; - Non-partnered: never married, divorced, separated, widowed. |
| English as 2^nd^ language | Binary (yes/no). Self-reported English as not 1^st^ language to learn to speak as a child. |
| Employment status | Binary (employed/unemployed or not in the labour force). Self-reported employment status. |
| Household regular income | Continuous. Household gross regular income.  “Household gross regular income, positive values” subtracted by “Household financial year gross regular income, negative values”. Missing values have been imputed by HILDA data owner.  The income model did not include redundancy and severance payments, inheritance or bequests, irregular transfers from non-resident parents or non-household members, lump sum workers' compensation, or other irregular payments. A comprehensive explanation of the income model, detailing regular, irregular, and total income estimates, has been previously published elsewhere [4]. |
| Social support | Binary (low/medium-to-high). The social support scores were calculated based on a ten-item scale.[5] Low social support was defined by an average score below 4.69.[6] |
| Major life events | Binary (yes/no). Self-reporting any of the following events in the past 12 months: birth/adoption of new child, death of a close friend, death of close relative/family member, death of spouse or child, major worsening in finances, fired or made redundant, serious injury/illness to family member, serious personal injury/illness, close family member detained in jail, detained in jail, changed jobs, got married, changed residence, victim of a property crime, pregnancy, promoted at work, got back together with spouse, retired from the workforce, separated from spouse, victim of physical violence.  The event not included due to none of p-value in univariate analyses to test association with insomnia, depression, and utility scores lower than 0.25 was major improvement in finances. |
| Diet quality | Binary (low/high). Diet quality scores (ranging from 0-31 points) were calculated based on 19 questions about consumption of nutrient-dense foods, fruits and vegetables, type of milk, salt added to meals, discretionary foods, and frequency of purchasing food from an outlet. A full description of how to calculate the diet scores was published elsewhere.[7] Low diet quality was defined by a cut-off point of 21 scores or below.[7] |
| Physical activity | Binary (low frequency/high frequency). Self-reported frequency of participating moderate-to-intensive physical activity for at least 30 minutes.   - Low frequency: 3 times a week or less - High frequency: more than 3 times a week |
| Smoking | Binary (yes/no). Self-reported current smoking on a 5-point scale:   - No: Never smoked or former smoke - Yes: Current smoker |
| Alcohol use | Binary (high-risk/low-risk). Self-reported drinking alcohol on an 8-point scale   - High-risk: 5 days per week or more - Low-risk: Less than 5 days per week |
| Life changed by Covid | Categorical (great/moderate/a little/not at all). Self-reported extent of life changed because of the coronavirus crisis on a 4-point scale. |
| BMI: Body mass index; HILDA: Household, Income, and Labour Dynamics in Australia; K10: Kessler Psychological Distress Scale-10; MHI-5: Mental Health Inventory-5; SF-6D: Short Form-36 | |

# Table S2. Unweighted versus weighted mean of utility scores

| **Subgroup** | **Unweighted mean** | **Weighted mean** |
| --- | --- | --- |
| ***All*** | ***0.639 (0.246)*** | ***0.636 (0.247)*** |
| No insomnia and depression | 0.728 (0.181) | 0.726 (0.182) |
| Insomnia only | 0.623 (0.213) | 0.625 (0.215) |
| Depression only | 0.447 (0.234) | 0.446 (0.232) |
| Comorbid insomnia and depression | 0.314 (0.253) | 0.317 (0.254) |

# Table S3. Missing values in the analytical sample

| **Variable** | **Wave 13**  **N (%)** | **Wave 17**  **N (%)** | **Wave 21**  **N (%)** |
| --- | --- | --- | --- |
| Utility score | 0 (0.00) | 646 (6.26) | 733 (7.10) |
| Insomnia | 54 (0.52) | 357 (3.46) | 555 (5.38) |
| Depression – K10 | 8 (0.08) | 320 (3.10) | 506 (4.90) |
| Depression – MHI-5 | 106 (1.03) | 471 (4.56) | 559 (5.41) |
| Age | 0 (0.00) | 0 (0.00) | 0 (0.00) |
| Sex | 0 (0.00) | 0 (0.00) | 0 (0.00) |
| Serious physical illnesses | 0 (0.00) | 0 (0.00) | 0 (0.00) |
| Obesity | 310 (3.00) | 599 (5.80) | 626 (6.10) |
| Chronic pain | 0 (0.00) | 0 (0.00) | 0 (0.00) |
| Education | 5 (0.05) | 5 (0.05) | 5 (0.05) |
| Partnered | 0 (0.00) | 1 (0.01) | 3 (0.03) |
| English as 2^nd^ language | 1 (0.00) | 1 (0.00) | 1 (0.00) |
| Employment status | 0 (0.00) | 0 (0.00) | 0 (0.00) |
| Income | 0 (0.00) | 0 (0.00) | 0 (0.00) |
| Social support | 167 (1.60) | 535 (5.20) | 616 (6.00) |
| Major life events | 266 (2.60) | 546 (5.30) | 624 (6.00) |
| Diet quality | 738 (7.10) | 1,081 (10.50) | 1,300 (12.60) |
| Physical activity | 19 (0.20) | 371 (3.60) | 473 (4.60) |
| Smoking | 40 (0.40) | 400 (3.90) | 498 (4.80) |
| Alcohol use | 62 (0.60) | 416 (4.00) | 487 (4.70) |
| Life changed by Covid |  |  | 6 (0.10) |

# Table S4. Prevalence and utility scores across all definitions of depression at baseline (Wave 13)

|  | **Wave 13** | | | | | | | | |
| --- | --- | --- | --- | --- | --- | --- | --- | --- | --- |
| **Definition of depression** | **MHI-5 ≤ 62** | | **MHI-5 ≤ 54** | | **MHI-5 ≤ 74** | | **K10 ≥ 20** | | |
| Sensitivity | 0.74^a^ | | 0.63^a^ | | 0.90^a^ | | 0.66^b^ | | |
| Specificity | 0.91^a^ | | 0.96^a^ | | 0.80^a^ | | 0.92^b^ | | |
| **Measure** | **Prevalence** | **Utility score** | **Prevalence** | **Utility score** | **Prevalence** | **Utility score** | **Prevalence** | **Utility score** | |
| **No insomnia and depression** | **6,998 (68.8%)** | **0.736 (0.177)** | **7,543 (74.2%)** | **0.725 (0.185)** | **5,542 (54.5%)** | **0.768 (0.157)** | **7,145 (69.6%)** | **0.735 (0.179)** | |
| **Insomnia only** | **1,151 (11.3%)** | **0.647 (0.212)** | **1,419 (14.0%)** | **0.609 (0.221)** | **737 (7.2%)** | **0.699 (0.184)** | **1,201 (11.7%)** | **0.628 (0.218)** | |
| **Depression only** | **1,184 (11.6%)** | **0.451 (0.231)** | **639 (6.3%)** | **0.397 (0.225)** | **2,640 (26%)** | **0.554 (0.226)** | **1,114 (10.9%)** | **0.471 (0.236)** | |
| Mild |  |  |  |  |  |  | 656 (6.4%) | 0.514 (0.227) | |
| Moderate |  |  |  |  |  |  | 271 (2.6%) | 0.451 (0.224) | |
| Severe |  |  |  |  |  |  | 187 (1.8%) | 0.348 (0.237) | |
| **Comorbid insomnia and depression** | **833 (8.2%)** | **0.341 (0.254)** | **565 (5.6%)** | **0.277 (0.252)** | **1,247 (12.3%)** | **0.406 (0.261)** | **803 (7.8%)** | **0.342 (0.263)** | |
| Insomnia and mild depression |  |  |  |  |  |  | 325 (3.2%) | 0.454 (0.232) | |
| Insomnia and moderate depression |  |  |  |  |  |  | 213 (2.1%) | 0.354 (0.227) | |
| Insomnia and severe depression |  |  |  |  |  |  | 265 (2.6%) | 0.194 (0.255) | |
| Prevalence of depression | 19.8% |  | 11.9% |  | 38.3% |  | 18.7% |  | |
| Prevalence of insomnia | 19.5% |  | 19.5% |  | 19.5% |  | 19.5% |  | |
| Prevalence of insomnia in individuals with depression | 41.3% |  | 45.6% |  | 32.1% |  | 41.9% |  | |
| ^a^Sensitivity and specificity to define individuals with major depression and dysthymia according to DSM-III-R criteria [2]  ^b^Sensitivity and specificity to define individuals with anxiety/affective disorders according to DSM-IV criteria [8] | | | | | | | | |  |

# Table S5. Multi-level modelling results with different definitions of depression

(Adjusted by age, sex, mental and physical health, demographics, lifestyle, and Covid impact)

|  | **Base case** | **SA1** | **SA2** | **SA3** |
| --- | --- | --- | --- | --- |
| Definition of depression | MHI-5 score ≤ 62 | MHI-5 score ≤ 54 | MHI-5 score ≤ 74 | K10 score ≥ 20 |
| **Fixed effects** |  |  |  |  |
| Intercept | 0.902  (0.888 – 0.916)^***^ | 0.895  (0.881 – 0.909)^***^ | 0.929  (0.916 – 0.943)^***^ | 0.911  (0.897 – 0.925)^***^ |
| Insomnia | -0.058  (-0.064 – -0.051)^***^ | -0.068  (-0.074 – -0.062) ^***^ | -0.042  (-0.050 – -0.034)^***^ | -0.063  (-0.070 – -0.057)^***^ |
| Depression | -0.210  (-0.216 – -0.203)^***^ | -0.239  (-0.247 – -0.231) ^***^ | -0.170  (-0.175 – -0.165)^***^ | -0.194  (-0.201 – -0.187)^***^ |
| Insomnia*Depression | -0.023  (-0.034 – -0.013)^***^ | -0.001  (-0.013 – 0.011) | -0.050  (-0.060 – -0.039)^***^ | -0.015  (-0.026 – -0.004)^**^ |
| Age (centring at 18) | -0.002  (-0.002 – -0.002)^***^ | -0.002  (-0.002 – -0.002) ^***^ | -0.002  (-0.002 – -0.002)^***^ | -0.002  (-0.002 – -0.002)^***^ |
| Sex (Female) | -0.021  (-0.027 – -0.016)^***^ | -0.022  (-0.027 – -0.016) ^***^ | -0.017  (-0.022 – -0.012)^***^ | -0.021  (-0.026 – -0.015)^***^ |
| Serious physical illnesses | -0.045  (-0.050 – -0.040)^***^ | -0.045  (-0.051 – -0.040) ^***^ | -0.045  (-0.050 – -0.040)^***^ | -0.045  (-0.051 – -0.040)^***^ |
| Obesity | -0.032  (-0.037 – -0.027)^***^ | -0.034  (-0.039 – -0.029) ^***^ | -0.033  (-0.039 – -0.028)^***^ | -0.03  (-0.036 – -0.025)^***^ |
| Chronic pain | -0.169  (-0.177 – -0.162)^***^ | -0.173  (-0.181 – -0.165) ^***^ | -0.173  (-0.181 – -0.165)^***^ | -0.172  (-0.180 – -0.164)^***^ |
| Education (Medium) | -0.008  (-0.014 – -0.002)^*^ | -0.006  (-0.012 – -0.000) ^*^ | -0.012  (-0.018 – -0.006)^***^ | -0.007  (-0.013 – -0.000)^*^ |
| Education (Low) | -0.007  (-0.014 – 0.001) | -0.008  (-0.016 – 0.000) | -0.012  (-0.019 – -0.004)^**^ | -0.005  (-0.014 – 0.003) |
| Non-partnered | -0.01  (-0.015 – -0.005)^***^ | -0.009  (-0.015 – -0.004) ^**^ | -0.013  (-0.018 – -0.008)^***^ | -0.011  (-0.017 – -0.006)^***^ |
| English as 2nd language | -0.005  (-0.014 – 0.004) | -0.005  (-0.015 – 0.004) | -0.005  (-0.014 – 0.004) | -0.003  (-0.012 – 0.006) |
| Unemployed or not in the labour force | -0.04  (-0.046 – -0.035)^***^ | -0.039  (-0.045 – -0.034) | -0.045  (-0.051 – -0.040)^***^ | -0.041  (-0.047 – -0.035)^***^ |
| Income ($10k's) | 0.000  (0.000 – 0.001)^***^ | 0.000  (0.000 – 0.001) ^***^ | 0.000  (0.000 – 0.001)^**^ | 0.000  (0.000 – 0.001)^***^ |
| Low social support | -0.05  (-0.055 – -0.045)^***^ | -0.06  (-0.065 – -0.055) ^***^ | -0.05  (-0.055 – -0.045)^***^ | -0.057  (-0.062 – -0.052)^***^ |
| Major life events | -0.024  (-0.028 – -0.020)^***^ | -0.023  (-0.027 – -0.019) ^***^ | -0.024  (-0.028 – -0.020)^***^ | -0.025  (-0.029 – -0.021)^***^ |
| Low diet quality | -0.012  (-0.017 – -0.007)^***^ | -0.014  (-0.019 – -0.009) ^***^ | -0.01  (-0.014 – -0.005)^***^ | -0.011  (-0.016 – -0.006)^***^ |
| Low frequency of physical activity | -0.045  (-0.049 – -0.040)^***^ | -0.046  (-0.050 – -0.041) ^***^ | -0.044  (-0.048 – -0.039)^***^ | -0.049  (-0.054 – -0.044)^***^ |
| Smoking | -0.017  (-0.024 – -0.010)^***^ | -0.022  (-0.029 – -0.015) ^***^ | -0.019  (-0.025 – -0.012)^***^ | -0.017  (-0.024 – -0.010)^***^ |
| High-risk alcohol use | 0.013  (0.007 – 0.020)^***^ | 0.013  (0.007 – 0.020) ^***^ | 0.011  (0.005 – 0.018)^**^ | 0.013  (0.007 – 0.020)^***^ |
| Life changed by Covid (Great extent) | -0.009  (-0.020 – 0.001) | -0.013  (-0.024 – -0.002) ^*^ | -0.007  (-0.017 – 0.003) | -0.011  (-0.022 – -0.001)^*^ |
| Life changed by Covid (Moderate extent) | 0.004  (-0.006 – 0.014) | 0.001  (-0.009 – 0.011) | 0.006  (-0.004 – 0.016) | 0.002  (-0.008 – 0.012) |
| Life changed by Covid (Little extent) | 0.004  (-0.006 – 0.014) | 0.003  (-0.008 – 0.013) | 0.006  (-0.004 – 0.016) | 0.003  (-0.007 – 0.013) |
| **Random effect** |  |  |  |  |
| Level-2 variance | 0.019 | 0.020 | 0.019 | 0.024 |
| Level-1 variance | 0.010 | 0.011 | 0.011 | 0.036 |
| **Summary** |  |  |  |  |
| No. participants | 10,038 | 10,057 | 10,038 | 10,324 |
| No. observations | 25,390 | 25,553 | 25,390 | 29,593 |
| Marginal R-squared | 0.472 | 0.447 | 0.452 | 0.000 |
| Conditional R-squared | 0.655 | 0.639 | 0.651 | 0.599 |
| *P < 0.05, **P <0.01, ***P <0.001; SA: Sensitivity analysis | | | | |

# Table S6. Marginal mean differences in utility scores and effect size Cohen’s d of pairwise comparisons between subgroups defined by insomnia and depression

| **Subgroups defined by insomnia and depression** | | **Base case** | | | **SA1** | | | **SA2** | | | **SA3** | | |
| --- | --- | --- | --- | --- | --- | --- | --- | --- | --- | --- | --- | --- | --- |
|  |  | **MHI-5 score ≤ 62** | | | **MHI-5 score ≤ 54** | | | **MHI-5 score ≤ 74** | | | **K10 score ≥ 20** | | |
| **Group 1** | **Group 2** | **MMD (SE)** | **Cohen’s d (SE)** | | **MMD (SE)** | **Cohen’s d (SE)** | | **MMD (SE)** | **Cohen’s d (SE)** | | **MMD (SE)** | **Cohen’s d (SE)** | |
| No insomnia/ depression | Insomnia only | 0.058 (0.003) | 0.420 (0.024) | * | 0.068 (0.003) | 0.493 (0.023) | * | 0.042 (0.004) | 0.299 (0.029) | * | 0.063 (0.003) | 0.449 (0.024) | * |
| No insomnia/ depression | Depression only | 0.210 (0.003) | 1.530 (0.025) | *** | 0.239 (0.004) | 1.735 (0.032) | *** | 0.170 (0.003) | 1.224 (0.020) | *** | 0.194 (0.004) | 1.376 (0.026) | *** |
| No insomnia/ depression | Comorbid insomnia and depression | 0.291 (0.004) | 2.120 (0.031) | *** | 0.308 (0.005) | 2.237 (0.035) | *** | 0.261 (0.004) | 1.879 (0.027) | *** | 0.272 (0.004) | 1.932 (0.031) | *** |
| Depression only | Comorbid insomnia and depression | 0.081 (0.004) | 0.591 (0.033) | ** | 0.069 (0.006) | 0.502 (0.041) | ** | 0.091 (0.004) | 0.655 (0.026) | ** | 0.078 (0.005) | 0.556 (0.033) | ** |
| MMD: Marginal mean difference of group 1 versus group 2; SE: standard error  Higher utility scores denote better quality of life.  *: small effect (Cohen’s d>=0.2); **: medium effect (Cohen’s d>=0.4); ***: large effect (Cohen’s d>=0.8) | | | | | | | | | | | | | |

# Table S7. Multi-level modelling results with depression severity defined by K10

(Adjusted by age, sex, mental and physical health, demographics, lifestyle, and Covid impact)

|  | **Subgroup analysis**  Depression defined by K10  β (95% CI) |
| --- | --- |
| **Fixed effects** |  |
| Intercept | 0.911 (0.897 – 0.924) |
| Insomnia | -0.064 (-0.070 – -0.057) |
| Mild depression | -0.154 (-0.163 – -0.146) |
| Moderate depression | -0.232 (-0.245 – -0.220) |
| Severe depression | -0.305 (-0.319 – -0.290) |
| InsomniaMild depression | 0.019 (0.005 – 0.033) |
| InsomniaModerate depression | 0.027 (0.009 – 0.045) |
| InsomniaSevere depression | -0.023 (-0.042 – -0.005) |
| Age (centring at 18) | -0.002 (-0.002 – -0.002) |
| Sex (Female) | -0.019 (-0.025 – -0.014) |
| Serious physical illnesses | -0.044 (-0.050 – -0.039) |
| Obesity | -0.03 (-0.035 – -0.024) |
| Chronic pain | -0.167 (-0.175 – -0.159) |
| Education (Medium) | -0.006 (-0.012 – -0.000) |
| Education (Low) | -0.006 (-0.014 – 0.002) |
| Non-partnered | -0.008 (-0.014 – -0.003) |
| English as 2nd language | -0.004 (-0.013 – 0.005) |
| Unemployed or not in the labour force | -0.037 (-0.043 – -0.032) |
| Income ($10k's) | 0.000 (0.000 – 0.001) |
| Low social support | -0.049 (-0.054 – -0.044) |
| Major life events | -0.025 (-0.029 – -0.021) |
| Low diet quality | -0.01 (-0.015 – -0.005) |
| Low frequency of physical activity | -0.047 (-0.052 – -0.043) |
| Smoking | -0.013 (-0.020 – -0.006) |
| High-risk alcohol use | 0.014 (0.008 – 0.021) |
| Life changed by Covid (Great extent) | -0.012 (-0.023 – -0.002) |
| Life changed by Covid (Moderate extent) | -0.001 (-0.011 – 0.009) |
| Life changed by Covid (Little extent) | 0.000 (-0.010 – 0.010) |
| **Random effect** |  |
| Level-2 variance | 0.019 |
| Level-1 variance | 0.010 |
| **Summary** |  |
| No. participants | 10,057 |
| No. observations | 25,553 |
| Marginal R-squared | 0.468 |
| Conditional R-squared | 0.657 |

# Table S8. Marginal mean differences in utility scores and effect size Cohen’s d of pairwise comparisons between subgroups defined by insomnia and depression severity levels

| **Subgroups defined by insomnia and depression** | | **Subgroup analyses** | | |
| --- | --- | --- | --- | --- |
|  |  | **Depression defined by K10** | | |
| **Group 1** | **Group 2** | **MMD (SE)** | **Cohen’s d (SE)** | |
| No insomnia and depression | Insomnia only | 0.064 (0.003) | 0.462 (0.024) | * |
| No insomnia and depression | Mild depression only | 0.154 (0.004) | 1.121 (0.031) | *** |
| No insomnia and depression | Comorbid insomnia and mild depression | 0.199 (0.006) | 1.444 (0.041) | *** |
| No insomnia and depression | Moderate depression only | 0.232 (0.006) | 1.688 (0.046) | *** |
| No insomnia and depression | Comorbid insomnia and moderate depression | 0.269 (0.007) | 1.955 (0.051) | *** |
| No insomnia and depression | Severe depression only | 0.305 (0.007) | 2.212 (0.053) | *** |
| No insomnia and depression | Comorbid insomnia and severe depression | 0.392 (0.006) | 2.843 (0.048) | *** |
| Mild depression only | Comorbid insomnia and mild depression | 0.044 (0.007) | 0.322 (0.048) | * |
| Moderate depression only | Comorbid insomnia and moderate depression | 0.037 (0.009) | 0.267 (0.064) | * |
| Severe depression only | Comorbid insomnia and severe depression | 0.087 (0.009) | 0.631 (0.064) | ** |
| MMD: Marginal mean difference of group 1 versus group 2; SE: standard error  Higher utility scores denote better quality of life  *: small effect (Cohen’s d>=0.2); **: medium effect (Cohen’s d>=0.4); ***: large effect (Cohen’s d>=0.8) | | | | |

**References**

1. Norman, R., Viney, R., Brazier, J., Burgess, L., Cronin, P., King, M., Ratcliffe, J., & Street, D. (2014). Valuing SF-6D Health States Using a Discrete Choice Experiment. Med Decis Making, 34(6), 773-786.

2. Cuijpers, P., Smits, N., Donker, T., ten Have, M., & de Graaf, R. (2009). Screening for mood and anxiety disorders with the five-item, the three-item, and the two-item Mental Health Inventory. Psychiatry Res, 168(3), 250-255.

3. Australian Bureau of Statistics. (2012). Use of the Kessler Psychological Distress Scale in ABS Health Surveys, Australia, 2007-08 Retrieved Mar, 2023, from <https://www.abs.gov.au/ausstats/abs@.nsf/Lookup/4817.0.55.001Chapter92007-08>

4. Wilkins, R. (2014). HILDA PROJECT TECHNICAL PAPER SERIES No. 1/14, March 2014. Derived Income Variables in the HILDA Survey Data: The HILDA Survey ‘Income Model’ Australia: Melbourne Institute of Applied Economic and Social Research, .

5. Reynolds, J., & Aletraris, L. (2007). Work–Family Conflict, Children, and Hour Mismatches in Australia. Journal of Family Issues, 28(6), 749-772.

6. Milner, A., Krnjacki, L., & LaMontagne, A. D. (2016). Age and gender differences in the influence of social support on mental health: a longitudinal fixed-effects analysis using 13 annual waves of the HILDA cohort. Public Health, 140, 172-178.

7. Oftedal, S., Rayward, A. T., Fenton, S., & Duncan, M. J. (2021). Sleep, diet, activity, and incident poor self-rated health: A population-based cohort study. Health Psychology, 40(4), 252-262.

8. Andrews, G., & Slade, T. (2001). Interpreting scores on the Kessler Psychological Distress Scale (K10). Aust N Z J Public Health, 25(6), 494-497.
